# Pericentromeric Transcription of Novel Pathogen-Related Human GPS Genes in Cancers is Regulated by C19MC miRNAs, CEBPB, IFN-γ, and IFN-β

**DOI:** 10.21203/rs.3.rs-8621807/v1

**Published:** 2026-02-04

**Authors:** Goodwin Jinesh, Isha Godwin, Marco Napoli, Elsa Flores, Andrew Brohl

**Affiliations:** Moffitt Cancer Center

**Keywords:** GPS: Genes at Pericentromeric-repeat Sequences, Pericentromeric transcription, C6GPS, C17GPS, CEBPB-LAP, Nonsense-mediated decay (NMD), IFN-γ, IFN-β, Plasmodium ovale wallikeri, Staphylococcus hominis, Streptococcus pneumoniae, Streptomyces sp., Miniopterus natalensis, Vibrio vulnificus, Salmonella enterica, C19MC, miR-519D, miR-520G, miR-526B

## Abstract

Pericentromeric transcription is unique to testis, and oocytes among the normal tissues. However, its regulation in cancer is not well-understood. Here, we discover a novel human, intron-less, coding, pericentromeric GPS gene family in cancer cells, with protein-level homology to microbial proteins from *Plasmodium*, *Staphylococcus, Streptococcus*, and *Mycobacterium tuberculosis*. GPS proteins harbor a conserved FPFP-motif, characteristic of a *Mycobacterial* protein that hijacks the host ERK-1/2 phosphorylation. We examined the two most expressed GPS family genes (*C6GPS*, and *C17GPS*) in cancer cells and discovered that the pericentromeric transcription is regulated by interferon-γ and interferon-β, CEBPB-LAP, and antiviral C19MC-miRNAs. Furthermore, GPS mRNAs are suppressed by truncation mutations, and nonsense-mediated decay (NMD). Thus, we discovered a novel pathogen-related GPS gene family in the human genome, and its pericentromeric transcription-regulatory network. This discovery will help to understand the role of GPS pericentromeric transcription in the biology, immunotherapy, and host-pathogen relationships of cancers in the future.

## Summary

Pericentromeric transcription is unique to testis, and oocytes among the normal tissues. However, its regulation in cancer is not well-understood. Here, we discover a novel human, intron-less, coding, pericentromeric GPS gene family in cancer cells, with protein-level homology to microbial proteins from, and. GPS proteins harbor a conserved FPFP-motif, characteristic of a protein that hijacks the host ERK-1/2 phosphorylation. We examined the two most expressed GPS family genes (, and) in cancer cells and discovered that the pericentromeric transcription is regulated by interferon-γ and interferon-β, CEBPB-LAP, and antiviral C19MC-miRNAs. Furthermore, GPS mRNAs are suppressed by truncation mutations, and nonsense-mediated decay (NMD). Thus, we discovered a novel pathogen-related GPS gene family in the human genome, and its pericentromeric transcription-regulatory network. This discovery will help to understand the role of GPS pericentromeric transcription in the biology, immunotherapy, and host-pathogen relationships of cancers in the future.

## Introduction

Human centromeres, and pericentromeres are highly enriched with repetitive sequences^1–5^, and the presence of coding genes within these regions is rare. Although centromeres, and pericentromeres constitute a large portion of the non-coding region within the human genome, transcription of non-coding RNA genes and a few coding genes does happen within centromeric, and pericentromeric regions^6,7^ resulting in clearly defined functions such as meiosis^8–10^, mitosis^11^, self-renewal in senescent cells^1^, centromeric cohesion^12^, CENP-A targeting to the centromere^13^, and drug resistance^14^. Therefore, centromeric and pericentromeric transcription is important for the chromosome dynamics during cell division^12^, and can serve as a basic mechanism related to chromosomal instability or stability^15^. However, regulation of the human pericentromeric transcription is not well-understood except that it is repressed in most normal human tissues, excluding testis^7^, and mature oocytes^3^, indicating a developmental role, which was demonstrated in mice^16,17^ in addition to gametogenesis.

Pericentromeric chromatin differs from centromeric chromatin by having predominant H3K27me3 methylation mark through transcript-directed recruitment of methylation factors to the pericentromeric region^18^. H3K27me3 mark is also associated with PRC-1 and PRC-2-dependant chromatin compaction and heterochromatinization to repress genes^19^. Hypomethylation of pericentromeric chromatin leads to interferon (IFN) response^20^. Interestingly, the IFN-β promoter tends to associate with pericentromeric heterochromatin, and which dissociates from pericentromeric chromatin upon viral infection to promote IFN expression^21^. IFN signaling is tightly associated with the viral response of the host^22^, and viruses stimulate an antiviral response miRNA cluster from chromosome-19 (Chromosome-19 miRNA cluster: C19MC)^23–28^, which is widely expressed in human cancers with critical functions^19,29–32^. Importantly, we uncovered the role of C19MC in nuclear division without nuclear envelope breakdown (NEBD) during a novel meiosis-III that happens in multiple human cancers^29^. Of note, meiosis is also related to pericentromeric transcription^8–10^, testis, and oocyte development^33,34^. The biological context and regulation of pericentromeric transcription during C19MC viral response, and interferon immune responses are not understood to date.

Human pathogens (such as multiple viruses^35,36^, *Plasmodium*^37,38^, *Staphylococcus*^39,40^, *Streptococcus*^41–43^, *Mycobacterium tuberculosis*^44,45^, and *Salmonella*^46^) are capable of eliciting interferon response in hosts. IFN signals through STATs, and extracellular signal-regulated kinases-1 and 2 (popularly referred to as ERK-1/2) to activate CCAAAT/Enhancer-binding Protein-β (CEBPB)-dependent transcription^47^. CEBPB is often co-expressed with antiviral C19MC in human cancers^31^ and modulates transcription in response to C19MC miRNA/IFN-γ^25^ and has cooperative functions with C19MC miRNAs^28^. In the context of chronic/persistent infections pathogens disable IFN signaling at multiple level^36^, including the inhibition of ERK signaling. For example, the FPFP motif of the Mce3E protein of *Mycobacterium tuberculosis* binds to and inhibits host/human ERK signaling in the context of persistent *Mycobacterium tuberculosis* infection^48^. However, the relationship between human pathogens, interferon signaling, CEBPB, and C19MC miRNAs in the pericentromeric transcription context remains unknown.

Nonsense-mediated decay (NMD) is a mechanism of RNA catabolism where the unwanted transcripts such as mutated/translation truncated mRNAs are degraded using exonucleases, and endonucleases. Viruses^49^ and other pathogens influence the host NMD mechanism, or have their own NMD mechanism to remodel the transcriptome^50^. A widespread absence of pericentromeric transcripts in normal tissues^7^ suggests that either a strong transcriptional repression at the pericentromeric region such as heterochromatinization, or an RNA decay mechanism such as NMD, might suppress the pericentromeric transcripts, but this is not understood to date.

Here, we discovered and characterized a novel intron-less, coding, pericentromeric GPS (Genes at Pericentromeric-repeat Sequences) human gene family with protein-level homology to proteins from *Plasmodium ovale/walkeri*, *Staphylococcus hominis, Streptococcus pneumoniae, Streptomyces kurssanovii, Mycobacterium tuberculosis*, *Salmonella enterica*, and other pathogens, expressed in human cancer cells. We identify a highly conserved FPFP motif within the entire GPS gene family member proteins along with multiple proteins from various human pathogens, and a bat genus that often serves as a reservoir for viruses (*Miniopterus*). We further found that the pericentromeric transcription is regulated by IFN-γ, IFN-β, CEBPB-LAP, miR-519D, miR-520G, and miR-526B (C19MC-miRNAs). Finally, we uncovered that the pericentromeric GPS mRNA transcripts are suppressed by nonsense-mediated decay (NMD). Thus, our study sheds light on the role of GPS pericentromeric transcription in the biology of cancers, especially in the contexts of immune (interferons), antiviral response (C19MC miRNAs), transcription (CEBPB), mutation and NMD, and paves the way to better understand antiviral, pathogen-induced, and pericentromeric transcription-directed signaling in human host cells in future.

## Results

### Discovery and characterization of C6GPS, a pericentromeric intron-less gene

**Figure 1:**
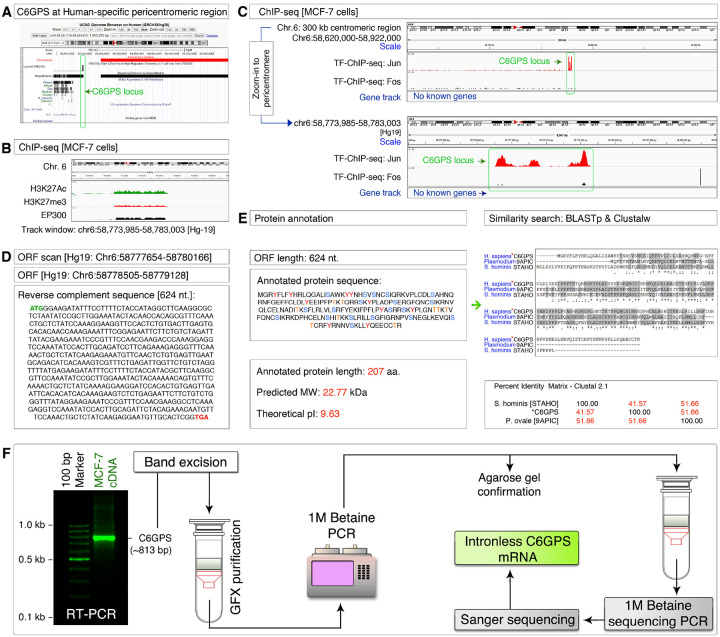
Discovery and characterization of pericentromeric intronless C6GPS gene **A**, Identification of a H3K27Ac region (would-be C6GPS locus) within pericentromeric repeats of human chromosome-6 unique to human genome among 100 vertebrates list of UCSC Genome Browser. Red bar: centromeric region. **B**, ChIP-seq data showing pericentromeric H3K27me3 mark and p300 binding along with the H3K27Ac mark at the would-be C6GPS region of chromosome-6. **C**, ChIP-seq data showing c-Jun binding at the would-be C6GPS region of chromosome-6. **D**, Identification of an open reading frame (ORF) within the H3K27Ac plus H3K27me3 mark on chromosome-6 pericentromeric region. **E**, Annotated protein sequence, characteristics and protein sequence similarity to proteins from *Plasmodium* and *Staphylococcus hominis*. **F**, RT-PCR amplification of C6GPS mRNA and its Sanger sequencing to identify that the C6GPS mRNA is an intronless transcript.

To understand pericentromeric transcription, we scanned the pericentromeric regions of the human genome for the H3K27ac mark using the UCSC genome browser. We identified a strong H3K27ac mark that falls within the repetitive DNA region but closely outside the centromere of the chromosome-6 at the p-arm side (Figure-1A). Notably, this region is not conserved and is specific to humans among the 100 vertebrate genomes of the PhyloP set (Figure-1A). We refer to this locus as the c*hromosome-*6 g*ene at the* p*ericentromeric* s*equence* (*C6GPS*) based on the findings below. To confirm the pericentromeric nature and transcription competent potential, we examined the MCF-7 ChIP-seq data of H3K27ac (transcription potential), p300 (transcription potential), and H3K27me3 (pericentromeric mark) and found that the *C6GPS* locus is indeed at the pericentromeric transcriptional region (Figure-1B). We chose the MCF-7 cell line for its known phenotypic features in meiosis-III^29^. We also found that c-Jun transcription factor can bind to the *C6GPS* locus in MCF-7 cells (Figure-1C). Considering multiple transcription factors (c-Jun and p300) can bind to the *C6GPS* locus, we examined this region for any potential open reading frames (ORF) and found an ORF of 624 nucleotides in length with start, and stop codons (Figure-1D). The annotated protein sequence of this ORF indicated that C6GPS is a 207 amino acid long protein with a predicted molecular weight of 22.77 kDa and an isoelectric pH of 9.63, enriched in tyrosine, serine, and threonine residues, suggesting that C6GPS could be regulated by both receptor tyrosine kinases (RTKs), and serine/threonine kinases (STKs) (Figure-1E). Sequence homology search using BLASTp has shown that C6GPS has strong similarities to pathogen proteins such as 9APIC of *Plasmodium falciparum*, and STAHO of *Staphylococcus hominis*, among many others (see below) (Figure-1E).

To determine if C6GPS is transcribed into mRNA, we performed RT-PCR in MCF-7 cells and found a feeble product of the expected size (~ 813 base pairs). To confirm its identity and to understand if it has undergone splicing, we reamplified this product and subjected it to Sanger sequencing (Figure-1F). The sequencing data revealed that *C6GPS* is an intronless gene and its mRNA is not subjected to splicing (Figure-1F and Figure-S1). Taken these data together, C6GPS is an intronless pericentromeric gene with protein-level homology to proteins from human pathogens, and is transcribed in MCF-7 cells without splicing.

### Discovery and characterization of C17GPS, a pericentromeric intron-less gene

**Figure 2:**
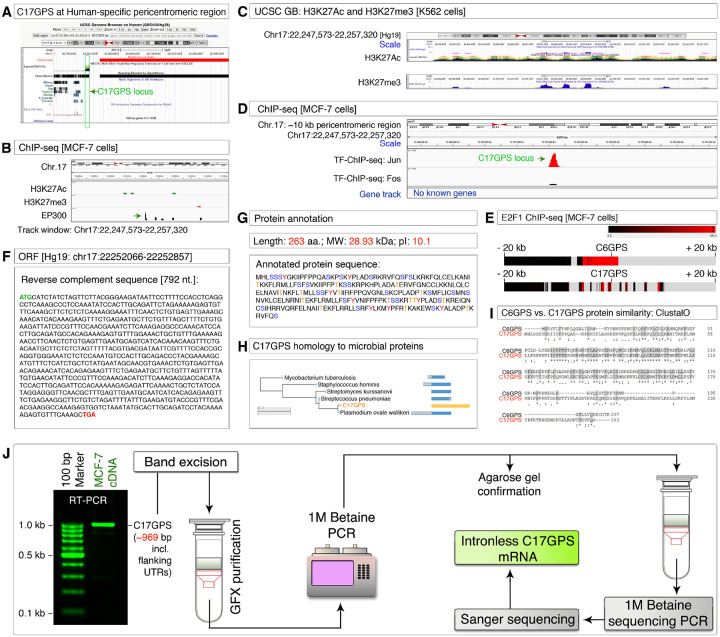
Discovery and characterization of pericentromeric intronless C17GPS gene **A**,Identification of a H3K27Ac region (would-be C17GPS locus) within pericentromeric repeats of human chromosome-17 unique to human genome among 100 vertebrates list of UCSC Genome Browser. Red bar: centromeric region. **B-C**, ChIP-seq data showing p300 binding at the would-be C17GPS region of chromosome-17 in MCF-7 cells (panel-B), and the presence of pericentromeric H3K27me3 mark along with the H3K27Ac mark of the corresponding C17GPS region of chromosome-17 in K562 cells (panel-C). **D**,ChIP-seq data showing c-Jun binding at the would-be C17GPS region of chromosome-17. **E**, ChIP-seq data showing E2F-1 binding at the C6GPS and would-be C17GPS loci of chromosome-6 and 17 respectively. The start codons are marked by gene symbols with 20 kb up and downstream regions marked. **F**, Identification of an open reading frame (ORF) within the H3K27Ac plus H3K27me3 mark on chromosome-17 pericentromeric region. **G**, Annotated protein sequence, characteristics of *C17GPS* gene. **H**, C17GPS protein sequence similarity to proteins from *Plasmodium ovale, Streptococcus pneumoniae, Mycobacterium tuberculosis, Streptomyces*, and *Staphylococcus hominis*. **I**, ClustalOmega: Protein sequence similarity between C6GPS and C17GPS. **J**,RT-PCR amplification of C17GPS mRNA and its Sanger sequencing to identify that the C17GPS mRNA is an intronless transcript.

To further investigate the pericentromeric transcription we undertook a nucleotide sequence-based search of C6GPS within the human genome and found no strong homologous genes. However, when we further examined the pericentromeric regions of the human genome for the H3K27ac mark, we identified another strong H3K27ac mark that falls within the repetitive DNA region but closely outside the centromere (pericentromeric region) of the chromosome-17 at the p-arm side (Figure-2A). Notably, this region is not conserved and is specific to humans among the 100 vertebrate genomes of the PhyloP set (Figure-2A). We refer to this locus as the c*hromosome-*17 g*ene at the* p*ericentromeric* s*equence* (*C17GPS*) based on the findings below. To confirm the pericentromeric nature and transcription competent potential, we examined the MCF-7 ChIP-seq data of H3K27ac (transcription potential), p300 (transcription potential), and H3K27me3 (pericentromeric mark) and found that the *C17GPS* locus is indeed bound by p300 but had feeble H3K27ac and H3K27me3 marks in MCF-7 cells (Figure-1B). However, the K562 cell line exhibited strong H3K27ac and H3K27me3 marks, indicating that the *C17GPS* locus is in a potentially transcription-competent pericentromeric region (Figure-1C). We also found that c-Jun transcription factor can bind to the *C17GPS* locus in MCF-7 cells (Figure-1D). We examined both *C6GPS* and *C17GPS* loci for E2F1 binding (a meiosis-promoting transcription factor) and found that E2F1 can bind to both genes (Figure-1E). We examined the *C17GPS* locus for any potential open reading frames (ORF) and found an ORF of 792 nucleotides length with start, and stop codons (Figure-1F). The annotated protein sequence from this ORF indicated that C17GPS is a 263 amino acid long protein with a predicted molecular weight of 28.93 kDa and an isoelectric pH of 10.1, enriched in tyrosine, serine, and threonine residues suggesting that C17GPS could be regulated by both receptor tyrosine kinases (RTKs), and serine/threonine kinases (STKs) (Figure-1G). Sequence homology search using BLASTp has shown that the N-terminal half of C17GPS has strong similarities to pathogen proteins of *Plasmodium ovale*, *Staphylococcus hominis, Mycobacterium tuberculosis, Streptococcus pneumoniae, and Streptomyces*, among many others (see below) (Figure-1H). Though our C6GPS nucleotide-based search did not identify C17GPS, their proteins had strong conserved motifs indicating the existence of a protein-level homology despite having a low homology at the nucleotide-level (Figure-2I).

To determine if *C17GPS* is transcribed into mRNA, we performed RT-PCR in MCF-7 cells and obtained a feeble product of the expected size (~ 969 base pairs). To confirm its identity and to understand if it has undergone splicing, we reamplified this product and subjected it to Sanger sequencing (Figure-1J). The sequencing data revealed that *C17GPS* is also an intronless gene and its mRNA is not subjected to splicing (Figure-1J and Figure-S2). Taken these data together, C17GPS is an intronless pericentromeric gene with protein-level homology to proteins from human pathogens, and is transcribed in MCF-7 cells without splicing.

### Discovery and characterization of pathogen-related GPS family of pericentromeric intron-less genes: the conserved FPFP motif and its truncation in cancer cells

**Figure 3:**
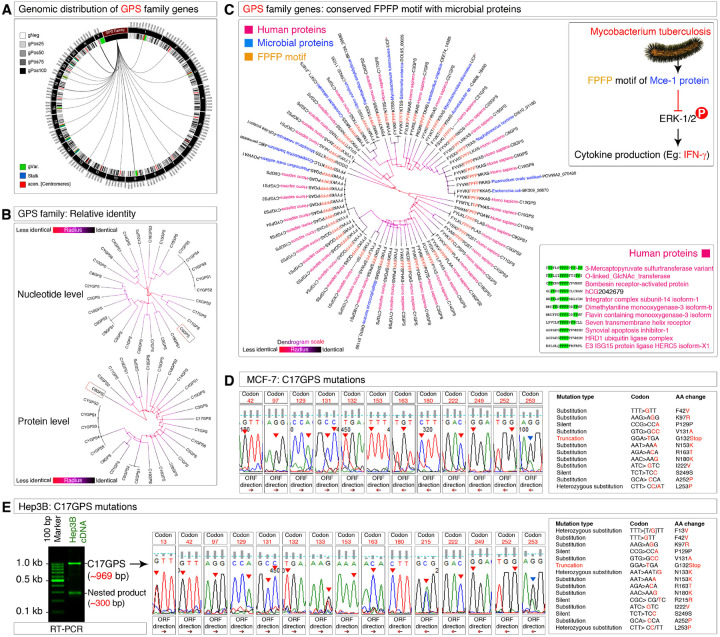
Discovery of GPS gene family, conserved FPFP-motif and its truncation by mutations **A**, Circos plot showing the genomic distribution of the GPS family genes across human genome. Note the predominant localization of these genes close to centromeres (red). **B**, Nucleotide level (Top) and protein level (Bottom) homology among the GPS gene/protein family members. The radial distance from center (C6GPS) represents the identity level from C6GPS (Boxed). **C**, The GPS family proteins have conserved FPFP motif with flanking region homology to various microbial and a mammalian vertebrate (Miniopterus: bat) and is shown in radial cladogram. The bottom right inset represents the FPFP motif homology to RefSeq proteins from human proteome. The top right inset represents the known ERK-1/2 inhibitory function of FPFP motif from *Mycobacterium tuberculosis* Mce-1 protein. **D**, Sanger sequence-based mutations identified in MCF-7 C17GPS mRNA compared to the reference genome. Individual codons were shown with arrows indicating the ORF reading direction. A summary of mutations is indicated in the inset table on right. **E**, For additional confirmation C17GPS mRNA from Hep3B cell line was sequenced and a summary of similar and unique mutations were included in the table on right. The ~300 bp RT-PCR product was also sequenced and confirmed as the nested product of C17GPS. Note that the truncation mutation by stop codon introduction was similar in both MCF-7 cells (Panel-D) and Hep3B cells (Panel-E).

Identification of C6GPS and C17GPS at the pericentromeric region of different chromosomes prompted us to search for additional similar genes within the human genome. A nucleotide sequence-based search of C17GPS within the human genome resulted in the identification of 27 other homologous intronless genes, all located at the pericentromeric region of human chromosomes, with the exception of two genes that are located at the non-pericentromeric regions of chromosome-9 (Figure-3A). Notably, we did not identify pericentromeric genes at chromosomes-4, 13, and 22 based on *C17GPS* sequence similarity search (Figure-3A). We named these genes based on the chromosomes in which they are located, for example if the gene is localized to chromosome-1, then we named it as *C1GPS*, and so on. At this point, we called these genes collectively as the “GPS gene family”. Chromosome-Y harbors 5 *GPS* genes (*CYGPS1–5*) which are identical in sequence and located close to each other suggesting that this could be due to the result of repetitive DNA expansion (Figure-3B). On the other hand, *C9GPS1* and *C9GPS2* were also identical but not located at the pericentromeric repeats (Figure-3B). At the nucleotide level, all GPS gene family genes showed considerable homology except C6GPS (Figure-3B). All wild-type nucleotide sequences of the ORFs of the GPS gene family members are provided in Supplementary table-1. At the protein level, about half of the GPS gene family members shown close homology to C17GPS (Figure-3B). All wild-type protein sequences of the GPS gene family members are provided in Supplementary table-2. Again, C6GPS stood out as different among all the GPS family members (Figure-3B) despite having conserved motifs with C17GPS (Figure-2I).

Conserved peptide motif analysis among all GPS gene family members revealed the presence of a conserved 12–13 amino acid sequence. Homology search of this sequence using BLASTp revealed that this motif is also conserved with the proteins from multiple human pathogens including *Plasmodium ovale, Mycobacterium tuberculosis, Staphylococcus hominis, Escherichia coli, Vibrio sp., Salmonella sp., Acinetobacter sp., Cronobacter sp., Lactobacillus crispatus*, and others (Figure-3C). While this stretch of 12–13 amino acid sequence is well conserved, an FPFP motif within this sequence is notable as its function is known in the case of *Mycobacterium tuberculosis* FPFP motif of Mce3E protein, which binds to and inhibits host/human ERK phosphorylation-based signaling in the context of persistent infection^48^ (Figure-3C). Among the human proteins, the FPFP motif is also present in a handful of proteins (Figure-3C), notably in human chorionic gonadotrophin (hCG), a known meiosis stimulator^29^.

We next investigated the potential role of the FPFP motif of GPS genes in cancer. Sanger sequencing of C17GPS mRNA from MCF-7 cells revealed multiple mutations compared to the UCSC human reference genome, ranging from silent, substitution, to truncation mutations (Figure-3D). The truncation mutation identified was at codon G132Stop, which could potentially result in the loss of FPFP motif from the translation product (Figure-3D). To check if these mutations are specific to MCF-7 cells or also present in other cancer cells, we examined C17GPS mRNA in Hep3B cells. C17GPS mRNA of Hep3B cells harbored identical (codons F42V, K97R, P129P silent, V131A, N153K, R163T, N180K, I222V, S249S silent, A252P, and L253P) as well as unique (F13V, N133K, and R215R silent) mutations compared to the MCF-7 cell line (Figure-3E). Of note, these alterations could be due to *de novo* mutations or polymorphisms and understanding of which requires population-based studies. Importantly, the truncation mutation was identical to the MCF-7 C17GPS mRNA (G132Stop) (Figure-3E). Thus, the truncation mutation resulting in the loss of FPFP motif of GPS genes in cancer could be a common mechanism.

Taken together, these data demonstrate that GPS genes are a family of pericentromeric intronless genes located in most human chromosomes with a homologous FPFP ERK-1/2 inhibitory motif at their protein sequences, which are lost due to truncation mutations in MCF-7 and Hep3B cell lines.

### Antiviral C19MC miRNAs are expressed with pericentromeric GPS genes in the Interferon context

**Figure 4:**
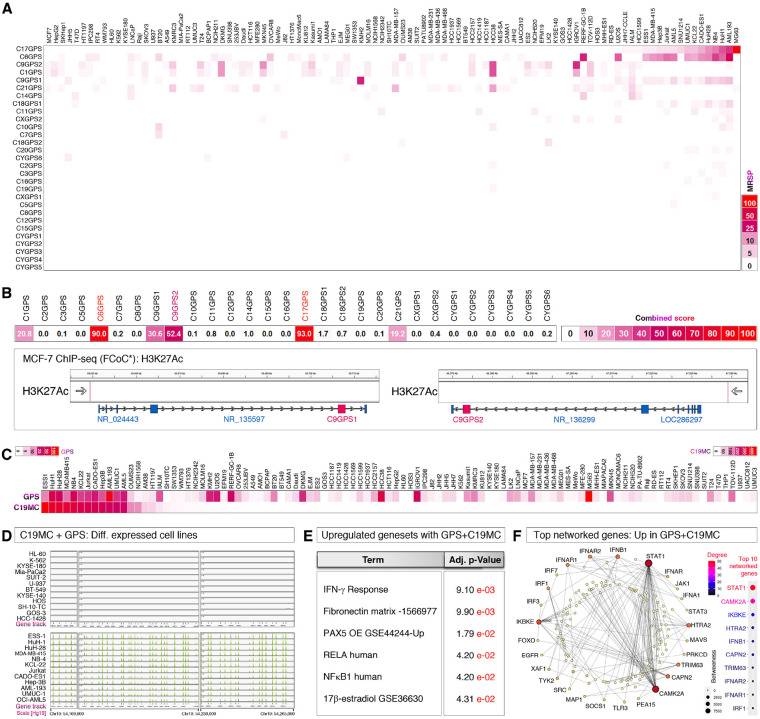
Pericentromeric transcription of GPS gene family matches with C19MC expression and interferonsignaling **A**, Examination of GPS gene family mRNAs in 100 CCLE cell lines from multiple cancer types. **B**, Combined mRNA expression score representation of GPS genes. Note that the C6GPS and C17GPS are the widely and strongly expressed mRNAs among all GPS genes. C9GPS1 and C9GPS2 despite express good levels of mRNAs (Top panel), these are not pericentromeric but located as part of non-coding RNA genes as host genes [Bottom panels: MCF-7 H3K27Ac ChIP-seq data of fold change over control (*FCoC)]. **C**, CCLE 100 cell line RNA-seq and miRNA-seq integrated data set analysis for co-expression of GPS gene family mRNAs with antiviral C19MC miRNAs. Sorted based on C19MC miRNA expression. **D**, Selection of cell line groups (n=12 each) that express both GPS genes and C19MC miRNAs or lack expression of both classes of RNAs: based on panel-C. The C19MC expression (Green peaks) is visualized in IGV. **E**, EnrichR analysis of differentially upregulated genes in C19MC+GPS RNA expressing cells showing interferon-γ pathway involvement. **F**,NetworkAnalyst analysis of highly networked interferon pathway genes from differentially upregulated genes in C19MC+GPS RNA expressing cells (Network) showing STAT-1 and IRF-1 as most networked transcription factors (Dot plot on right).

Considering the role of the FPFP motif in ERK-1/2 signaling and the role of ERK-1/2 in IFN-γ production, an essential role of interferons in the GPS gene family expression is conceivable. ERK-1/2 regulate the transcription of IFN-γ through the transcription factor CCAAAT/Enhancer-binding Protein-β (hereafter referred to as CEBPB)^47^ in addition to its other targets, and IFN-γ is involved in the antiviral immunity^51,52^. Furthermore, multiple human pathogenic viruses are capable of eliciting C19MC miRNA response^26,27^ as well as interferon response in hosts^35,36^. Therefore, we evaluated whether GPS mRNAs co-express with C19MC miRNAs. For this purpose, we first evaluated the expression of GPS gene mRNAs in 100 human cancer cell lines and found that GPS gene mRNAs are widely expressed in human cancer cell lines (Figure-4A). C6GPS and C17GPS mRNAs were the most expressed GPS family genes, followed by C9GPS1 and C9GPS2 mRNAs, which are not in fact pericentromeric genes but driven by non-coding RNA (ncRNA) host genes (Figure-4B). We matched the 100 cell line GPS gene expression data with C19MC miRNA data (miRNA-seq) and found that C19MC expression is tightly associated with GPS mRNA expression (Figure-4C). However, GPS gene mRNAs were also expressed in a small subset of cell lines without C19MC miRNA expression suggesting that the GPS gene family mRNAs could also be regulated independent of C19MC miRNA expression context.

To understand the signaling context between the co-expression of C19MC miRNAs and GPS gene family mRNAs, we performed differential gene expression profiling of RNA-seq data of cell lines that co-express both GPS mRNAs plus C19MC miRNAs versus cells that do not express both RNAs (Figure-4C–D). While the results are enriched with interferon-related genes, we identify that the IFN-γ response geneset, and STAT-1/IRF1 as the most significantly enriched as well as most networked transcription factors (Figure-4E–F).

### Interferons, C19MC miRNAs, all-trans retinoic acid (ATRA), and NMD regulate pericentromeric transcription and mRNA levels of the GPS family genes

**Figure 5:**
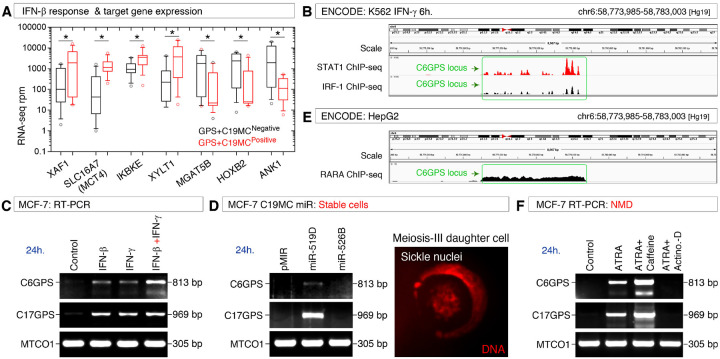
Interferons, C19MC miRNAs, all-trans retinoic acid (ATRA), and NMD regulate pericentromerictranscription of GPS family genes **A**, Differential expression of IFN-β response and target genes in GPS+C19MC versus GPS+C19MC^Negative^ cell lines. *Significant (p-value <0.05). **B**, ChIP-seq data showing STAT-1 and IRF-1 binding at the C6GPS region of chromosome-6 in K562 cells. **C**, Reverse transcriptase PCR showing interferons (IFN-γ + IFN-β) co-operate to promote pericentromeric transcription of *C6GPS* and *C17GPS* genes. **D**, Reverse transcriptase PCR showing C19MC miRNA-519D promotes pericentromeric transcription of *C6GPS* and *C17GPS* genes (Left panel) which correlates with meiotic sickle nuclear phenotype shown after live staining the DNA with Hoechst-33342 (Right panel). **E**, ChIP-seq data showing RARA binding at the C6GPS region of chromosome-6 in HepG2 cells. **F**, Nonsense-mediated decay (NMD) of C6GPS and C17GPS pericentromeric transcript RNAs were overcome by meiosis-inducer all-trans retinoic acid (ATRA) by transcription (Actinomycin-D inhibits it). Caffeine, a NMD inhibitor further augments the mRNA levels.

Many of the IFN-γ pathway genes are also related to the IFN-β pathway, which is also involved in antiviral response, and were upregulated in C19MC plus *GPS* gene expression positive cell lines (Figure-5A). Some of the top downregulated genes were also a direct target of IFN-β regulated genes: for example, Ankyrin repeat gene *ANK1* product is a target for IKBKE kinase, which usually targets ankyrin repeats (Figure-5A). We examined two transcription factors, STAT-1 and IRF-1 binding to make sure interferon-regulated transcription factors can bind to the *C6GPS* locus using MCF-7 ChIP-seq data. Both STAT-1 and IRF-1 can bind to the *C6GPS* locus (Figure-5B). Therefore, we examined if IFN-γ and IFN-β could modulate the pericentromeric transcription of *C6GPS* and *C17GPS* in MCF-7 cells. The results revealed that both IFN-γ and IFN-β at 1 nM final concentrations can induce the pericentromeric transcription of both *C6GPS* and *C17GPS* genes at 24 hours, and their combination had an additive effect on *C6GPS* gene transcript compared to *C17GPS* (Figure-5C). This result suggested that both IFN-γ and IFN-β could use independent as well as overlapping pathways to regulate pericentromeric transcription to achieve the additive effect.

Next, we examined the effect of stable overexpression of antiviral C19MC miRNAs in MCF-7 cells. While we attempted three individual C19MC miRNAs (miR-519D, miR-520G, and miR-526B), we could generate stable cells only for miR-519D, and miR-526B. The C19MC miR-519D strongly induced the pericentromeric transcription of *C17GPS* compared to the *C6GPS* gene, whereas miR-526B did not have any effect (Figure-5D). This correlated with the sickle nuclear meiosis-III phenotype (Figure-5D), which we identified previously in response to miR-519D in MCF-7 cells^29^.

As all-trans retinoic acid, a stimulator of meiosis-III in cancer cells^23^, which signals through one of its many receptors RAR-α (RARA), we examined the available RARA-ChIP-seq data to find whether RAR-α can bind to the *C6GPS* locus and found that RAR-α binds to the *C6GPS* locus in HepG2 cells (Figure-5E). Usage of all-trans retinoic acid (ATRA) in MCF-7 cells induced the pericentromeric transcription of both *C6GPS* and *C17GPS* mRNAs, and the usage of nonsense-mediated decay (NMD) inhibitor caffeine with ATRA further increased the levels of *C6GPS* and *C17GPS* mRNAs, indicating that the pericentromeric mRNAs are also subjected to NMD (Figure-5F). The inability of ATRA to induce pericentromeric *C6GPS* and *C17GPS* mRNAs in the presence of transcription inhibitor actinomycin-D indicated that fresh transcription is involved in the induction of pericentromeric transcripts by ATRA as a single agent (Figure-5F).

Taken together, these results demonstrate that the pericentromeric transcription is regulated by IFN-γ, IFN-β, antiviral C19MC miRNAs, and ATRA in the meiosis-III context, and that these transcripts are subjected to nonsense-mediated decay.

### IFN-γ, CEBPB-LAP, and C19MC miRNAs cooperate to regulate pericentromeric GPS gene transcription

**Figure 6:**
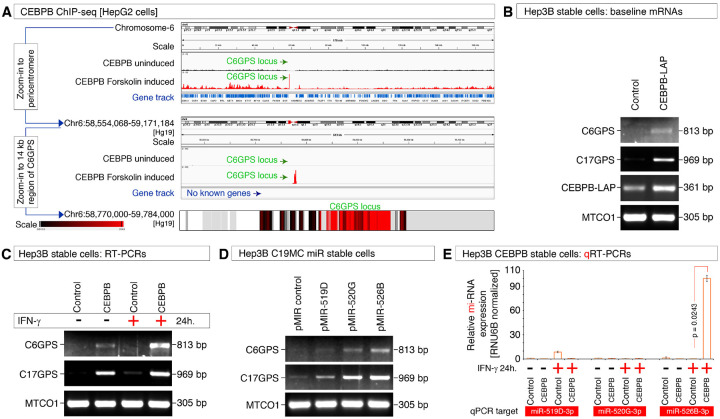
IFN-γ, CEBPB-LAP, and C19MC miRNAs cooperate to regulate pericentromeric GPS gene transcription **A**, ChIP-seq data (histogram peaks) showing the inducible CEBPB binding at the C6GPS region as the strongest binding locus of chromosome-6 in HepG2 cells. C6GPS zoom in locus was shows as heatmap. **B**, RT-PCR in CEBPB-LAP stablyoverexpressed Hep3B cells showing the baseline transcription of C6GPS and C17GPS mRNAs. **C**, RT-PCR in CEBPB-LAP stably overexpressed and control Hep3B cells showing the IFN-γ-induced augmented transcription of C6GPS and to a lesser extent C17GPS mRNAs. **D**, RT-PCR in antiviral C19MC miRNA stablyoverexpressed Hep3B cells (miR-519D, miR-520G, miR-526B) showing the baseline transcription of C6GPS and C17GPS mRNAs. **E**, qRT-PCR in CEBPB-LAP stably overexpressed and control Hep3B cells showing the IFN-γ-induced ~100-fold augmented transcription of endogenous antiviral C19MC miR-526B. Controls are set to the value of 1.

CEBPB liver-enriched activator protein (CEBPB-LAP isoform) modulates the transcriptional outcome of *MYO18B* gene by IFN-γ in the context of C19MC miRNAs in the liver context^25^. Therefore, we examined if CEBPB can bind to the *C6GPS* locus in HepG2 ChIP-seq data. CEBPB binding to the *C6GPS* locus is induced by CEBPB activating stimulus (Forskolin) and is the strongest among all CEBPB binding sites in the entire chromosome-6 (Figure-6A). Stable overexpression of CEBPB-LAP isoform in Hep3B cells (Hep3B cells express basal C19MC miRNAs, and GPS gene expression: Figure-4C–D, and lack basal IFN-γ expression^25^) by itself induced the pericentromeric transcription of *C6GPS* and *C17GPS* mRNAs (Figure-6B). As Hep3B cells lack basal expression of IFN-γ but not its receptors^25^, we examined the effect of exogenous IFN-γ in pericentromeric transcription. 1 nM IFN-γ in CEBPB-LAP stably overexpressed cells boosted the pericentromeric transcription of *C6GPS* and to a lesser extent of *C17GPS* genes (Figure-6C). Furthermore, stable overexpression of individual C19MC miRNAs miR-519D, miR-520G, and miR-526B induced the pericentromeric transcription of *C17GPS* and to a lesser extent *C6GPS* genes (Figure-6D).

Finally, we asked the question whether the IFN-γ induced hyper-pericentromeric transcription of C6GPS in CEBPB-LAP overexpressed cells involve C19MC miRNAs. Quantitative real-time PCR analysis of C19MC miRNAs in this context revealed that an approximately 100-fold induction of endogenous miR-526B (and possibly more miRNAs from the C19MC) over the basal expression is accompanied with the hyper-pericentromeric transcription of *C6GPS* in CEBPB-LAP overexpressed cells in IFN-γ treated condition (Figure-6E). Therefore, we conclude that the CEBPB-LAP transcription factor cooperates with IFN-γ, and C19MC miRNAs to induce pericentromeric transcription.

## Discussion

**Figure 7:**
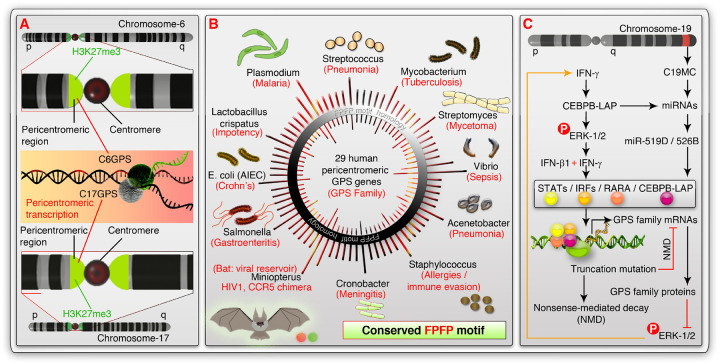
Regulation of pericentromeric transcription of GPS family genes, and their FPFP-motif microbial context **A**,Schematic showing the pericentromeric transcription of C6GPS and C17GPS genes from the chromosomes-6 and 17 respectively. The pericentromeric marker H3K27me3 is shown in green shade. **B**, Protein-level FPFP-motif homology of 29 GPS gene family members to various proteins of multiple pathogenic microbes and viral reservoir bats (*Miniopterus natalensis*). The microbes were illustrated and their associated disease conditions are mentioned in red font. **C**, Schematic showing the regulation of GPS family gene mRNAs by interferons, transcription factors like STAT-1, IRF-1, CEBPB-LAP, RARA, and how mutations trigger nonsense-mediated decay (NMD) of these mRNAs. The proposed function of GPS family protein FPFP-motifs in the inhibition of ERK-1/2 is also shown which is hampered by NMD. A circuit of antiviral C19MC miRNAs fitting this context is also indicated.

Cancers are known for rapid proliferation, which is mediated by mitotic cell division. Meiosis is confined to gametogenic tissues such as testis and ovary. However, non-germ line cancer cells from multiple cancer types exhibit spermatogenesis gene expression signature and exhibit a novel meiosis-III in the context of antiviral C19MC miRNA expression^29^. Pericentromeric transcription is restricted to testis^7^, and matured oocytes^3^ and is repressed in other normal tissues. Pericentromeric transcription is associated with IFN signaling^20^ and the IFN-β promoter tends to associate with pericentromeric heterochromatin, which dissociates from pericentromeric chromatin upon viral infection to promote IFN expression^21^. Here, we discovered a pericentromeric intron-less GPS gene family (Figure-7A–B) and identified that the pericentromeric transcription of these genes is regulated by cooperative interactions of IFN-γ, IFN-β, CEBPB-LAP, and C19MC miRNAs (Figure-7C).

Homology of GPS family protein FPFP motifs to various proteins from microbes, including *Plasmodium*, and *Mycobacterium tuberculosis*, sheds more insight on the functions of GPS family genes (Figure-7B). For example, *Plasmodium*-driven malaria is resisted by sickle cell disease and bacterial coinfections^53–55^, and the sickling pattern is strikingly similar to meiotic sickle cell-like morphological features upon miR-519D overexpression in MCF-7 cells^29^ (Figure-5D). Induction of pericentromeric transcription by meiotic inducer ATRA further supports this result (Figure-5E–F). Mycobacterium tuberculosis Mce3E protein FPFP motif is implicated in the ERK-1/2 phosphorylation^48^ (Figure-7C). The truncation mutation in the *C6GPS*, and *C17GPS* genes in cell lines can potentially remove the FPFP motif in these gene products. However, any other GPS gene products could still contribute to the FPFP motif-mediated influence of ERK-1/2 signaling. The level of FPFP motif and its flanking sequence homology to various human pathogenic microbial proteins to GPS family proteins (Figure-7B) indicates the importance of pericentromeric transcription of GPS genes. Although the GPS genes are not conserved among 100 vertebrate genomes at the nucleotide level (Figures-1A and 2A), their FPFP motif homology to proteins from the bat *Miniopterus* suggests a protein level conservation could exist among vertebrates. FPFP motif homology in *Miniopterus* is consistent with the fact that this bat is a reservoir for human pathogenic viruses^56^ (Figures-3C and 7B). *Miniopterus natalensis* bats are found in South Africa^57^, a place prone to HIV-1 epidemics^58^. *Miniopterus natalensis* bats have a mysterious chimera of CCR5 and CCR2 chemokine genes responsible for HIV-1 infection/entry^59^. This scenario is further complicated by increased maternal death associated with recent pregnancy (C19MC miRNAs express during pregnancy) by *Mycobacterium tuberculosis* in South Africa^60^. To add more spice, C19MC antiviral miRNA expression is strongly associated with HIV-infection signature in human cancers^29^. Therefore, the human GPS gene family and its homologous proteins of *Miniopterus natalensis, Mycobacterium tuberculosis* might play an important role in HIV-infection, and cancer biology in the context of C19MC miRNAs and interferons.

While the truncation mutation precluding the translation of FPFP motifs from C6GPS and C17GPS, unusual stop codons are also capable of triggering nonsense-mediated decay (NMD) of target RNAs. As expected, we found that the pericentromeric mRNAs of *C6GPS*, and *C17GPS* are undergoing NMD (Figure-5F). Therefore, this could be a mechanism to keep these mRNAs in check, or a mechanism to prevent the inhibition of ERK-1/2, as cancers heavily depend on ERK-1/2 for oncogene-driven tumorigenesis^61^.

In summary, our study uncovers a novel family of pericentromeric GPS genes that are related to various human pathogenic microbes, and their regulation by a cooperative network involving IFN-γ, IFN-β, CEBPB-LAP, and C19MC miRNAs. Further studies will help to understand the role of GPS pericentromeric transcription in the biology, immunotherapy, and host-pathogen relationships of cancers in the future.

## Materials and Methods

### Cell lines: culture and authentication by DNA fingerprinting.

Human Hep3B hepatocellular carcinoma cells (ATCC # HB-8064), and MCF-7 (HTB22) human breast cancer cells were cultured in MEM containing L-Glutamine and Sodium bi-carbonate (Sigma #M4655), with 10% FBS (Sigma#F0926), vitamins (Gibco Life Technologies #11120052), sodium pyruvate (Gibco Life Technologies #11360070), non-essential amino acids (Gibco Life Technologies #11140050), and penicillin-streptomycin (Gibco Life Technologies #15140122). The cells were identity confirmed by STR fingerprinting as per institutional/lab standards. Fresh revived cells were used after every 6 months or after ~25 passages. The cells in culture were periodically tested for mycoplasma using MycoAlert Kit (Lonza).

### Plasmids: C19MC miRNAs, CEBPB-LAP, and their controls

Glycerol stocks of mammalian expression vectors such as pMIR-CMV (Control), pMIR-CMV-519D (CR215546), pMIR-CMV-520G (CR215781), and pMIR-CMV-526B (CR215142) were purchased from Vigene Biosciences (Rockville, MD USA) and described previously^24^. The control pLenti-GIII-CMV-RFP-2A-Puro (Cat# LV084) and the LAP isoform-CEBPB pLenti-GIII-CMV-human-CEBPB-RFP-2A-Puro (Cat# LV796074) vectors were purchased from Applied Biological Materials Inc., Richmond, BC, Canada and described previously^25^. All plasmids were isolated using Qiagen MIDI prep kit (#12143) as per manufacturer’s instructions.

### Stable cell line generation and authentication

Hep3B stable cells with C19MC miRNA overexpression (miR-519D, miR-520G, and miR-526B) is described previously^24^. Briefly, the transfections were done using plasmids (not viruses) and Lipofectamine 2000 (Life Technologies # 11668019) and selected using 4 mg/ml puromycin (Invitrogen # A1113803) for 2 months while GFP/RFP positive clones were picked, expanded and frozen. MCF-7 stable cells with C19MC miRNA overexpression (miR-519D, and miR-526B) is generated as described above but with gradual increase in puromycin, where the miR-520G failed to grow as cell line after transfection, and the cells were sorted for GFP positivity instead of clone picking. The FACS sorted cells were further subjected to STR fingerprinting to make sure the identity of MCF-7 cells and to rule out cross contamination of cells.

Hep3B stable cells with CEBPB-LAP and its control plasmid overexpression is described previously^25^. Briefly, the cells were transfected using plasmids (not viruses) and Lipofectamine 2000 (Life Technologies # 11668019) as per manufacturer’s protocol, and selected using 4 mg/ml puromycin (Invitrogen # A1113803) for 2 months before colony picking by RFP positivity. The overexpression of CEBPB-LAP was confirmed by LAP-specific RT-PCR (See below for primer details).

### Reagents and treatment doses

#### Kits:

High-Capacity cDNA Reverse Transcription kit (ABI, Cat.# 4368814), Plasmid isolation MIDI-prep kit (Qiagen, Cat.# 12143), and Illustra GFX PCR DNA and Gel Band Purification Kit (GE Healthcare, Cat.# 28903470), Trizol RNA isolation reagent (ThermoFisher Scientific, Cat.# 15596018), miRNeasy Mini Kit (50) (Qiagen, Cat.# 217004).

#### Cytokines and treatment conditions:

IFN-γ (R&D Systems, Cat.# 285-IF-100, 1nM / 17 ng/ml for 24 h.), IFN-β (R&D Systems, Cat.# 8499-IF-010, 1nM / 20 ng/ml for 24 h.).

#### Chemical reagents and treatment conditions:

All-trans retinoic acid (ATRA) (Cayman Chemicals, Cat.# 11017, 1mM for 24 h.), betaine (5M stock: Sigma # B0300–1VL, St. Louis, MO, USA), and Caffeine (Cayman Chemicals, Cat.# 14118, 10mM for 24 h.).

### RT-PCR and sequencing primers

**Table:** 

Target mRNA	Primer sequence	Annealing
C6GPS-Forward*†	5’- TGCTCTATCAAGAGAAATGTTCCACC-3’	60°C
C6GPS-Reverse*†	5’- GAAAAGGGAATATCTTTCCATAAAAGG-3’	60°C
C17GPS-Forward*	5’- CAACGAGAGTTTCCAAAGTGCTCTC-3’	60°C
C17GPS-Reverse*	5’- GGGATAACTGCACCTAACTACACGG-3’	60°C
MTCO1-Forward**	5’-ATGAGCTGGAGTCCTAGGCACAGC-3’	60°C
MTCO1-Reverse**	5’-AACCTGTTCCTGCTCCGGCCTCC-3’	60°C
CEBPB-LAP-Forward**	5’-AACGCCTGGTGGCCTGGGACCC-3’	60°C
CEBPB-LAP-Reverse**	5’-AAGAGGTCGGAGAGGAAGTCGTGG-3’	60°C

* Sequencing & RT-PCR primers used in this study;

** Published previously^25^;

† G.G.J personal order.

### RNA isolation and Reverse transcriptase PCRs

Total RNAs were isolated from cells were isolated from cells using either Trizol reagent, or using miRNeasy kit with RNAse-free DNAse digestion step as per manufacturer’s instructions. 20 ml complementary DNA (cDNA) synthesis reactions were performed using 1000 ng RNA and High-Capacity cDNA Reverse Transcription Kit with 1.5M final concentration of betaine (from 5M stock). The temperature steps for cDNA synthesis were, 25°C for 10m, 37°C for 120m and 85°C for 5m. The cDNAs were further diluted with 30 ml of nuclease free water and then 2.5 ml was used for each PCR reaction. For PCR reactions 1M betaine (final conc.) was used along with regular PCR reaction components (**Per reaction:** 10X PCR buffer without MgCl_2_: 2.5 ml; 25 mM MgCl_2_: 1ml; 5M Betaine: 5 ml; dNTP mix [2.5 mM each]: 1 ml; Taq polymerase: 1.25 U; DEPC water: 12.5 ml). The primer sequences were indicated above and each primer (forward and reverse) are used at 1 ml per reaction from a 10 mM stock. All PCR reactions were subjected to an initial denaturation of 3 minutes, and cycling denaturation (95°C) time of 1-minute, annealing temperature of 60°C (30 seconds) and 1 minute of extension time (72°C), with 34 cycles. A final extension time of 5 minutes was given for complete product synthesis. The PCR reactions were run on 2% agarose gels with GeneRuler 100 bp DNA Ladder (ThermoFisher Scientific #SM0243). The gels were imaged using LI-COR Odyssey Fc imager (Lincoln, NE, USA). The expected product sizes were indicated in the figures.

### Sanger sequencing of C6GPS and C17GPS: mutation, and splicing analyses

Initial RT-PCR amplificons of C6GPS, and C17GPS were reamplified for Sanger sequencing purposes. The reamplified products were GFX-column purified as per manufacturer’s instructions, and 5–10ng of purified products were submitted to paired-end Sanger sequencing PCR reaction with 1M betaine and single primer (forward or reverse) at Azenta (Genewiz/Azenta, USA). The sequences were analyzed using FinchTV chromatogram reader for mutations and splicing by comparing the corresponding UCSC human genome hg19 build as reference (The coordinates are indicated in the figures). The following mutant sequences (compared to the reference genome sequence of the ORF) were submitted to GenBank: C6GPS of MCF-7 cells (Accession: PX444937), C17GPS of MCF-7 cells (Accession: PX444936), C17GPS of Hep3B cells (Accession: PX444938).

### Bioinformatic ORF identification of *C6GPS* and *C17GPS* genes, protein annotation

Pericentromeric human chromosome-6 and 17 regions were examined for H3K27Ac and H3K27me3 marks by enabling corresponding ChIP-seq layers in addition to repetitive DNA and centromeric DNA layers in UCSC genome browser (Hg19 build). DNA sequences that harbor H3K27Ac and H3K27me3 marks at the pericentromeric repetitive regions were subjected to 6-frame open reading frame analysis (ORF) in NCBI-ORF finder (https://www.ncbi.nlm.nih.gov/orffinder/) with ATG and alternative initiation codon, maximum ORF length, and standard genetic code options on. The ORFs were annotated to protein sequence using single letter amino acid code, to obtain calculated molecular weight using 110 Daltons weight for average amino acid.

### GPS gene epigenetic and transcription factor binding analysis: ChIP-seq

Transcription factor binding to GPS gene family loci (CEBPB, RARA, Jun, Fos, E2F1, EP300/p300, STAT-1 and IRF-1) and epigenetic histone regulatory marks (H3K27Ac, and H3K27me3) were examined using cell line ChIP-seq data. All ChIP-seq data were accessed from Encyclopedia of DNA Elements (ENCODE)^64^ or from UCSC Genome Browser (if indicated). CEBPB ChIP-seq data sets with or without forskolin induction in HepG2 cells [ENCODE: ENCSR000EEX file: ENCFF000XPP (fold change over control hg19) and ENCSR000BQI file: ENCFF321NDM (fold change over control hg19)] were examined for CEBPB binding at whole chromosome-6 as well as at the C6GPS locus and visualized using Integrative Genomics Viewer (IGV: BROAD institute, version 2.4.10) as peaks or as heatmap. The data range was kept constant (500) for both uninduced and forskolin induced peak tracks whereas the data range was represented as scale for heatmap. Other ChIP-seq data used were: RARA HepG2 (ENCSR500WXT: fold change over control; data range FC: 0–15), Jun MCF-7 (ENCFF513YRC: Signal p-Value; data range FC: 0–10, 0–30, 0–60), Fos MCF-7 (ENCFF950XOS: Signal p-Value; data range FC: 0–10, 0–30, 0–60), E2F1 MCF-7 (ENCFF000ZLB: signal; data range FC: 0–800), EP300 MCF-7 (ENCSR000BTR: fold change over control; data range FC: 0–5), H3K27Ac MCF-7 (ENCSR752UOD: fold change over control; data range FC: 0–5), H3K27me3 MCF-7 (ENCSR000EWP: fold change over control; data range FC: 0–5), STAT-1 K562: IFN-γ treated for 6 hours (ENCSR000EHJ: Signal p-Value; data range FC: 0–20), IRF-1 K562: IFN-γ treated for 6 hours (ENCSR000EGT: Signal p-Value; data range FC: 0–20), H3K27Ac K562 [from UCSC Genome Browser], and H3K27me3 K562 [from UCSC Genome Browser]. These ChIP-seq data sets were examined for binding at whole chromosome or C6GPS or C17GPS loci and visualized using Integrative Genomics Viewer (IGV: BROAD institute, version 2.4.10) as peaks or as heatmap.

### BLASTn and BLASTp homology search and nomenclature of GPS gene family members

The C6GPS ORF was subjected to human genome contig and RefSeq transcriptome BLASTn searches in NCBI site (https://blast.ncbi.nlm.nih.gov/Blast.cgi) and no considerable matches were found. A similar search for C17GPS ORF yielded multiple ORFs with significant homology to C17GPS and predominantly localized to human pericentromeric regions. These genes are named based on the chromosome number in which it is located (For example, C3 if it is located on chromosome-3), and numbered if more than one such genes are located at same chromosomes (For example, six genes located on chromosome-Y: CYGPS1–6).

Annotated protein sequences of GPS family members were subjected to non-redundant protein search across all proteins from multiple organisms including humans using BLASTp (https://blast.ncbi.nlm.nih.gov/Blast.cgi) and the matching reference protein sequences (RefSeq) were collected for further phylogenetic analysis (See below). A similar search and reference sequence collection was also done for nucleotide sequences of GPS family genes.

### GPS family gene mapping to human genome (Circos)

Genomic visualization of GPS family genes was done using Circa software (http://omgenomics.com/circa) as described previously^28^. Genomic coordinates of GPS genes were collected based on the ATG start position of each gene (Hg19) from UCSC genome browser and layered along with the coordinates for centromeres, and Giemsa positivity of DNA. The circos plot was then composited and labelled in Adobe Photoshop CS5.

### GPS family nucleotide and protein similarity distance analysis (iTOL)

Both nucleotide and protein BLAST matching sequences were subjected to sequence homology analysis using EMBL-EBI Clustal Omega (protein/nucleotide options) (https://www.ebi.ac.uk/jdispatcher/msa/clustalo?stype=protein). iTOL (https://itol.embl.de/). The cladograms were composited, and labelled in Adobe Photoshop CS5. The cladogram homology/identity scale is read based on the circular but not radial lines. A similar analysis, and compositing were done for FPFP motif matching proteins.

### GPS gene family mRNA and C19MC miRNA expression and differential gene expression analysis in CCLE cell lines:

100 cell lines that have miRNA-seq and RNA-seq datasets from Cancer Cell Line Encyclopedia (CCLE: https://sites.broadinstitute.org/ccle/) database were integrated by matching the cell lines, and examined for all GPS mRNA expressions in RNA-seq BAM files. Maximum reads at single point (MRSP) within the GPS gene ORF was considered as expression level. To get the expression level of an individual GPS gene, the reads of that gene across all 100 cell lines were added up to get a combined score. To get an expression level of overall GPS gene family, all reads of GPS family per cell line were added up to get a GPS score. The GPS score is then integrated with the cumulative C19MC miRNA expression and sorted based on C19MC expression before generating heatmap.

From these 100 CCLE cell lines, high C19MC+GPS RNA expressing 12 cell lines, and an equal number of cell lines that lack C19MC+GPS RNAs were grouped (of GPS+C19MC-RNA^Positive^ and GPS+C19MC-RNA^Negative^ groups) for differential gene expression analysis. The differentially expressed genes (>2 or <−2 log_2_ fold change) with p-value <0.05 were subjected to EnrichR analysis, and top-ranking gene sets were subjected to networked gene analysis using NetworkAnalyst web server (https://www.networkanalyst.ca/)^65^ to find the top ranking transcription factors. The analysis was performed using the SIGNOR 2.0 database of Signaling Network as described previously^66^. Briefly, the top network node was organized into a circular/bi/tripartite layout before exporting the image. The gene names were relabeled in Adobe Photoshop CS5 to have legibility.

Node tables were exported and the degree and betweenness scores were fed into the ggplot2 package in R to generate ranked dotplots to see the top-networked genes (Transcription factors were chosen).

R code:

> library(ggplot2)

> ggplot(#DataFrameName, aes(x = Xgroup, y = YGene)) + geom_point(aes(size = Betweenness, color = Degree)) + scale_color_gradientn(colours = c(“black”, “blue”, “magenta”, “red”), limits = c(0, 50))

#DataFrameName: file name.

The gene names in dotplots were relabeled in Adobe Photoshop CS5 to have legibility and color match.

### Microscopy

C19MC miR-519D overexpressed stable MCF-7 cells were live stained with Hoechst-33342 (10 nM for 20 minutes; Cayman Chemicals, # 15547) for DNA and the sickle nuclear patterns of meiosis-III daughter cells were imaged using Zeiss Observer.Z1 microscope equipped with Axiocam 503 mono (Zeiss) camera, and composited in Adobe Photoshop CS5 as described previously^29^.

### Quantitative real-time PCR (qRT-PCR) quantification of C19MC miRNA expression

Quantitative real-time PCRs (qRT-PCRs) for C19MC miRNAs were performed as described previously^29^. Briefly, RNAs were isolated from CEBPB-LAP overexpressed and their control stable cells (treated with or without 1 nM of IFN-γ for 24 hours) using miRNeasy Mini Kit, quantified using nanodrop. 250 ng RNAs were used for cDNA synthesis [using Multiscribe reverse transcriptase, RNAse inhibitor, 10X buffer, dNTPs, (TaqMan MicroRNA Reverse Transcription Kit: ABI, Cat # 4366596) and RT TaqMan Primers has-miR-519d-3p (ThermoFisher: Assay ID: 002403; Cat# 4427975), hsa-miR-520g-3p (ThermoFisher: Assay ID: 001121; Cat# 4427975), hsa-miR-526b-3p (ThermoFisher: Assay ID: 002383; Cat# 4427975), and RNU6B Control (ThermoFisher: Assay ID: 001093; Cat# 4427975)]. The cDNAs were further subjected to quantitative PCR reactions using corresponding PCR primers with probes and TaqMan Universal PCR Master Mix (Life Technologies Cat# 4324018).

Comparative Ct (DDCt) was used to calculate the relative expression of C19MC miRNAs to the control cells, after normalizing the values based on RNU6B. The RNU6B values were set as 1 and the relative fold changes in C19MC miRNA expression were plotted using GraphPad Prism software as bar graphs with SEM as error bars (v7.04; La Jolla, CA, USA). The plot was composited and labelled in Adobe Photoshop CS5.

### Statistical analyses

For EnrichR analysis, only statistically significant differentially expressed genes were included in the feed gene set, and the top signatures thus obtained with adjusted p-value below 0.05 were considered significant. For qRT-PCR bar-plots and other box-plots t-test statistical analysis was done using GraphPad Prism software (v7.04; La Jolla, CA, USA). The box-whisker plot is of 10–90 percentile type with 50% transparency for whisker data points. For GPS+C19MC positive and negative CCLE cell line groups, n=12 cell lines for GPS+C19MC-RNA^Positive^ and GPS+C19MC-RNA^Negative^ groups each was set based on C19MC miRNA expression set, and an equal number of negative cell lines were included to have equal statistical power. For ChIP-seq data the fold change over control data set was used if available or included the control with same track height settings. Throughout the study the Student’s T-test p-value of 0.05 was considered significant and are indicated with an asterisk (*) or with the p-value.

## Supplementary Material

Supplementary Files

This is a list of supplementary files associated with this preprint. Click to download.

- SupplementaryText10192025Highlightamended.docx
- TableS2.xlsx
- TableS1.xlsx
- FigureS1.jpg
- FigureS2.jpg

## References

[1] Wang J. Inhibition of activated pericentromeric SINE/Alu repeat transcription in senescent human adult stem cells reinstates self-renewal. Cell Cycle 10, 3016–3030, doi:10.4161/cc.10.17.17543 (2011). 21862875 PMC3218602

[2] Younger S. T. & Rinn J. L. Silent pericentromeric repeats speak out. Proceedings of the National Academy of Sciences 112, 15008–15009, doi:doi:10.1073/pnas.1520341112 (2015). PMC4679049 26582791

[3] Dobrynin M. A. Human pericentromeric tandemly repeated DNA is transcribed at the end of oocyte maturation and is associated with membraneless mitochondria-associated structures. Scientific Reports 10, 19634, doi:10.1038/s41598-020-76628-8 (2020). 33184340 PMC7665179

[4] Eymery A., Callanan M. & Vourc’h C. The secret message of heterochromatin: new insights into the mechanisms and function of centromeric and pericentric repeat sequence transcription. Int J Dev Biol 53, 259–268, doi:10.1387/ijdb.082673ae (2009). 19412885

[5] Giunta S. & Funabiki H. Integrity of the human centromere DNA repeats is protected by CENP-A, CENP-C, and CENP-T. Proc Natl Acad Sci U S A 114, 1928–1933, doi:10.1073/pnas.1615133114 (2017). 28167779 PMC5338446

[6] Saffery R. Transcription within a Functional Human Centromere. Molecular Cell 12, 509–516, doi:10.1016/S1097-2765(03)00279-X (2003). 14536089

[7] Eymery A. A transcriptomic analysis of human centromeric and pericentric sequences in normal and tumor cells. Nucleic Acids Research 37, 6340–6354, doi:10.1093/nar/gkp639 (2009). 19720732 PMC2770647

[8] Baumann C., Zhang X., Viveiros M. M. & De La Fuente R. Pericentric major satellite transcription is essential for meiotic chromosome stability and spindle pole organization. Open Biology 13, 230133, doi:doi:10.1098/rsob.230133 (2023). 37935356 PMC10645078

[9] Broering T. J. BRCA1 establishes DNA damage signaling and pericentric heterochromatin of the X chromosome in male meiosis. J Cell Biol 205, 663–675, doi:10.1083/jcb.201311050 (2014). 24914237 PMC4050732

[10] Khalil A. M. & Driscoll D. J. Epigenetic regulation of pericentromeric heterochromatin during mammalian meiosis. Cytogenet Genome Res 129, 280–289, doi:10.1159/000315903 (2010). 20606401 PMC3202914

[11] Robbins A. R. Inhibitors of histone deacetylases alter kinetochore assembly by disrupting pericentromeric heterochromatin. Cell Cycle 4, 717–726, doi:10.4161/cc.4.5.1690 (2005). 15846093

[12] Chen Y., Zhang Q., Teng Z. & Liu H. Centromeric transcription maintains centromeric cohesion in human cells. Journal of Cell Biology 220, doi:10.1083/jcb.202008146 (2021). PMC8065269 33881484

[13] Quénet D. & Dalal Y. A long non-coding RNA is required for targeting centromeric protein A to the human centromere. eLife 3, e26016, doi:10.7554/eLife.03254 (2014). 25117489 PMC4145801

[14] Kanne J. Pericentromeric Satellite III transcripts induce etoposide resistance. Cell Death Dis 12, 530, doi:10.1038/s41419-021-03810-9 (2021). 34031359 PMC8144429

[15] Smurova K. & De Wulf P. Centromere and Pericentromere Transcription: Roles and Regulation … in Sickness and in Health. Front Genet 9, 674, doi:10.3389/fgene.2018.00674 (2018). 30627137 PMC6309819

[16] Probst A. V. A strand-specific burst in transcription of pericentric satellites is required for chromocenter formation and early mouse development. Dev Cell 19, 625–638, doi:10.1016/j.devcel.2010.09.002 (2010). 20951352

[17] Rangasamy D., Berven L., Ridgway P. & Tremethick D. J. Pericentric heterochromatin becomes enriched with H2A.Z during early mammalian development. EMBO J 22, 1599–1607, doi:10.1093/emboj/cdg160 (2003). 12660166 PMC152904

[18] Hall L. E., Mitchell S. E. & O’Neill R. J. Pericentric and centromeric transcription: a perfect balance required. Chromosome Research 20, 535–546, doi:10.1007/s10577-012-9297-9 (2012). 22760449

[19] Jinesh G. G. & Brohl A. S. The genetic script of metastasis. Biol Rev Camb Philos Soc 95, 244–266, doi:10.1111/brv.12562 (2019). 31663259

[20] Rajshekar S. Pericentromeric hypomethylation elicits an interferon response in an animal model of ICF syndrome. Elife 7, doi:10.7554/eLife.39658 (2018). PMC6261255 30484769

[21] Josse T. Association of the interferon-beta gene with pericentromeric heterochromatin is dynamically regulated during virus infection through a YY1-dependent mechanism. Nucleic Acids Res 40, 4396–4411, doi:10.1093/nar/gks050 (2012). 22287632 PMC3378888

[22] Lercher A. Type I Interferon Signaling Disrupts the Hepatic Urea Cycle and Alters Systemic Metabolism to Suppress T Cell Function. Immunity 51, 1074–1087.e1079, doi:10.1016/j.immuni.2019.10.014 (2019). 31784108 PMC6926485

[23] Jinesh G. G. C19MC miRNA-520G induces SP100 antiviral gene transcription and inhibits melanin production in skin cutaneous melanoma. Genes Dis 11, 60–63, doi:10.1016/j.gendis.2023.02.047 (2024). 37588194 PMC10425800

[24] Jinesh G. G. Mutant p53s and chromosome 19 microRNA cluster overexpression regulate cancer testis antigen expression and cellular transformation in hepatocellular carcinoma. Sci Rep 11, 12673, doi:10.1038/s41598-021-91924-7 (2021). 34135394 PMC8209049

[25] Jinesh G. G. Regulation of MYO18B mRNA by a network of C19MC miRNA-520G, IFN-gamma, CEBPB, p53 and bFGF in hepatocellular carcinoma. Sci Rep 10, 12371, doi:10.1038/s41598-020-69179-5 (2020). 32704163 PMC7378193

[26] Delorme-Axford E., Sadovsky Y. & Coyne C. B. The Placenta as a Barrier to Viral Infections. Annual Review of Virology 1, 133–146, doi:10.1146/annurev-virology-031413-085524 (2014). 26958718

[27] Delorme-Axford E. Human placental trophoblasts confer viral resistance to recipient cells. Proc Natl Acad Sci U S A 110, 12048–12053, doi:10.1073/pnas.1304718110 (2013). 23818581 PMC3718097

[28] Jinesh G. CEBPB, C19MC, and Defective Autophagy Drive Novel Podosomal Belt to Macropinocytosis Transition, Lipid Accumulation, and HBV A-to-I RNA-editing. Communications Biology x (2025).

[29] Jinesh G. G. C19MC drives nucleolar invasion of mitochondria and meiotic nuclear division in human cancers. iScience 27, 111132, doi:10.1016/j.isci.2024.111132 (2024). 39563898 PMC11575172

[30] Setty B. A. The genomic landscape of undifferentiated embryonal sarcoma of the liver is typified by C19MC structural rearrangement and overexpression combined with TP53 mutation or loss. PLoS Genet 16, e1008642, doi:10.1371/journal.pgen.1008642 (2020). 32310940 PMC7192511

[31] Jinesh G. G., Flores E. R. & Brohl A. S. Chromosome 19 miRNA cluster and CEBPB expression specifically mark and potentially drive triple negative breast cancers. PLoS One 13, e0206008, doi:10.1371/journal.pone.0206008 (2018). 30335837 PMC6193703

[32] Jinesh G. G. & Brohl A. S. Classical epithelial-mesenchymal transition (EMT) and alternative cell death process-driven blebbishield metastatic-witch (BMW) pathways to cancer metastasis. Signal Transduction and Targeted Therapy 7, 296, doi:10.1038/s41392-022-01132-6 (2022). 35999218 PMC9399134

[33] Griswold M. D. Spermatogenesis: The Commitment to Meiosis. Physiological Reviews 96, 1–17, doi:10.1152/physrev.00013.2015 (2016). 26537427 PMC4698398

[34] Marteil G., Richard-Parpaillon L. & Kubiak J. Z. Role of oocyte quality in meiotic maturation and embryonic development. Reproductive Biology 9, 203–224, doi:10.1016/S1642-431X(12)60027-8 (2009). 19997475

[35] Stewart W. E. & Stewart W. E. Interferon inducers. The interferon system, 27–57 (1981).

[36] Rojas J. M., Alejo A., Martín V. & Sevilla N. Viral pathogen-induced mechanisms to antagonize mammalian interferon (IFN) signaling pathway. Cellular and Molecular Life Sciences 78, 1423–1444, doi:10.1007/s00018-020-03671-z (2021). 33084946 PMC7576986

[37] Artavanis-Tsakonas K. & Riley E. M. Innate Immune Response to Malaria: Rapid Induction of IFN-γ from Human NK Cells by Live Plasmodium falciparum-Infected Erythrocytes1. The Journal of Immunology 169, 2956–2963, doi:10.4049/jimmunol.169.6.2956 (2002). 12218109

[38] Pais T. F. Brain endothelial STING1 activation by < i > Plasmodium-sequestered heme promotes cerebral malaria via type I IFN response. Proceedings of the National Academy of Sciences 119, e2206327119, doi:doi:10.1073/pnas.2206327119 (2022). PMC9457060 36037380

[39] Tuffs S. W. Superantigens promote *Staphylococcus aureus* bloodstream infection by eliciting pathogenic interferon-gamma production. Proceedings of the National Academy of Sciences 119, e2115987119, doi:doi:10.1073/pnas.2115987119 (2022). PMC8872782 35165181

[40] Parker D. & Prince A. Staphylococcus aureus Induces Type I IFN Signaling in Dendritic Cells Via TLR9. The Journal of Immunology 189, 4040–4046, doi:10.4049/jimmunol.1201055 (2012). 22962685 PMC3466375

[41] Zangari T., Ortigoza Mila B., Lokken-Toyli Kristen L. & Weiser Jeffrey N. Type I Interferon Signaling Is a Common Factor Driving Streptococcus pneumoniae and Influenza A Virus Shedding and Transmission. mBio 12, 10.1128/mbio.03589-03520, doi:10.1128/mbio.03589-20 (2021). PMC8545127 33593970

[42] Parker D. Streptococcus pneumoniae DNA Initiates Type I Interferon Signaling in the Respiratory Tract. mBio 2, 10.1128/mbio.00016-00011, doi:10.1128/mbio.00016-11 (2011). PMC3101776 21586648

[43] Mitchell A. J. Inflammasome-Dependent IFN-γ Drives Pathogenesis in Streptococcus pneumoniae Meningitis. The Journal of Immunology 189, 4970–4980, doi:10.4049/jimmunol.1201687 (2012). 23071286

[44] Novikov A. Mycobacterium tuberculosis Triggers Host Type I IFN Signaling To Regulate IL-1β Production in Human Macrophages. The Journal of Immunology 187, 2540–2547, doi:10.4049/jimmunol.1100926 (2011). 21784976 PMC3159798

[45] Desvignes L., Wolf A. J. & Ernst J. D. Dynamic Roles of Type I and Type II IFNs in Early Infection with Mycobacterium tuberculosis. The Journal of Immunology 188, 6205–6215, doi:10.4049/jimmunol.1200255 (2012). 22566567 PMC3370955

[46] Bao S., Beagley K. W., France M. P., Shen J. & Husband A. J. Interferon-γ plays a critical role in intestinal immunity against Salmonella typhimurium infection. Immunology 99, 464–472, doi:10.1046/j.1365-2567.2000.00955.x (2000). 10712678 PMC2327174

[47] Hu J. ERK1 and ERK2 Activate CCAAAT/Enhancer-binding Protein-β-dependent Gene Transcription in Response to Interferon-γ *. Journal of Biological Chemistry 276, 287–297, doi:10.1074/jbc.M004885200 (2001). 10995751

[48] Fenn K., Wong C. T. & Darbari V. C. Mycobacterium tuberculosis Uses Mce Proteins to Interfere With Host Cell Signaling. Front Mol Biosci 6, 149, doi:10.3389/fmolb.2019.00149 (2019). 31998747 PMC6961568

[49] Leon K. & Ott M. An ‘Arms Race’ between the Nonsense-mediated mRNA Decay Pathway and Viral Infections. Seminars in Cell & Developmental Biology 111, 101–107, doi:10.1016/j.semcdb.2020.05.018 (2021). 32553580 PMC7295464

[50] Vembar S. S., Droll D. & Scherf A. Translational regulation in blood stages of the malaria parasite Plasmodium spp.: systems-wide studies pave the way. WIREs RNA 7, 772–792, doi:10.1002/wrna.1365 (2016). 27230797 PMC5111744

[51] Mendes-Monteiro L. & Viejo-Borbolla A. Using structure-function information from IFN-γ-binding proteins and biased agonists to uncouple immunostimulatory and immunosuppressive activities. Trends in Immunology 46, 284–294, doi:10.1016/j.it.2025.02.013 (2025). 40102163

[52] Samuel C. E. Antiviral Actions of Interferons. Clinical Microbiology Reviews 14, 778–809, doi:doi:10.1128/cmr.14.4.778-809.2001 (2001). 11585785 PMC89003

[53] Mudji J. E., Blum J., Rice T. D. & Baliraine F. N. Congenital malaria and neonatal bacterial co-infection in twins prematurely born to a mother with sickle-cell anaemia in the Democratic Republic of the Congo. Malariaworld J 8, 14 (2017). 34532237 PMC8415076

[54] Aidoo M. Protective effects of the sickle cell gene against malaria morbidity and mortality. The Lancet 359, 1311–1312, doi:10.1016/S0140-6736(02)08273-9 (2002). 11965279

[55] Friedman M. J. Erythrocytic mechanism of sickle cell resistance to malaria. Proceedings of the National Academy of Sciences 75, 1994–1997, doi:doi:10.1073/pnas.75.4.1994 (1978). PMC392469 347452

[56] Calisher C. H., Childs J. E., Field H. E., Holmes K. V. & Schountz T. Bats: Important Reservoir Hosts of Emerging Viruses. Clinical Microbiology Reviews 19, 531–545, doi:doi:10.1128/cmr.00017-06 (2006). 16847084 PMC1539106

[57] Junker K., Bain O. & Boomker J. Helminth parasites of Natal long-fingered bats, Miniopterus natalensis (Chiroptera: Miniopteridae), South Africa. Onderstepoort J Vet Res 75, 261–265 (2008). 19040141

[58] Puren A. J. The HIV-1 epidemic in South Africa. Oral Dis 8 Suppl 2, 27–31, doi:10.1034/j.1601-0825.2002.00007.x (2002). 12164655

[59] Fernandes A. P., Agueda-Pinto A., Pinheiro A., Rebelo H. & Esteves P. J. Evolution of CCR5 and CCR2 Genes in Bats Showed Multiple Independent Gene Conversion Events. Viruses 14, doi:10.3390/v14020169 (2022). PMC8877049 35215768

[60] Khan M., Pillay T., Moodley J. M., Connolly C. A. & Durban Perinatal T. B. H. I. V. S. G. Maternal mortality associated with tuberculosis-HIV-1 co-infection in Durban, South Africa. AIDS 15, 1857–1863, doi:10.1097/00002030-200109280-00016 (2001). 11579249

[61] Jinesh G. G., Sambandam V., Vijayaraghavan S., Balaji K. & Mukherjee S. Molecular genetics and cellular events of K-Ras-driven tumorigenesis. Oncogene 37, 839–846, doi:10.1038/onc.2017.377 (2018). 29059163 PMC5817384

[62] An integrated encyclopedia of DNA elements in the human genome. Nature 489, 57–74, doi:10.1038/nature11247 (2012). 22955616 PMC3439153

[63] Ghandi M. Next-generation characterization of the Cancer Cell Line Encyclopedia. Nature 569, 503–508, doi:10.1038/s41586-019-1186-3 (2019). 31068700 PMC6697103

[64] Consortium E. P. An integrated encyclopedia of DNA elements in the human genome. Nature 489, 57–74, doi:10.1038/nature11247 (2012). 22955616 PMC3439153

[65] Zhou G. NetworkAnalyst 3.0: a visual analytics platform for comprehensive gene expression profiling and meta-analysis. Nucleic Acids Research 47, W234–W241, doi:10.1093/nar/gkz240 (2019). 30931480 PMC6602507

[66] Jinesh G. G. & Godwin I. Filaggrin(High) melanomas exhibit active FGFR and allergic signatures with impaired GNA14 and Th1 signatures. Front Genet 16, 1569403, doi:10.3389/fgene.2025.1569403 (2025). 40765578 PMC12322895

